# A Review of Data Fusion Techniques

**DOI:** 10.1155/2013/704504

**Published:** 2013-10-27

**Authors:** Federico Castanedo

**Affiliations:** Deusto Institute of Technology, DeustoTech, University of Deusto, Avenida de las Universidades 24, 48007 Bilbao, Spain

## Abstract

The integration of data and knowledge from several sources is known as data fusion. This paper summarizes the state of the data fusion field and describes the most relevant studies. We first enumerate and explain different classification schemes for data fusion. Then, the most common algorithms are reviewed. These methods and algorithms are presented using three different categories: (i) data association, (ii) state estimation, and (iii) decision fusion.

## 1. Introduction

In general, all tasks that demand any type of parameter estimation from multiple sources can benefit from the use of data/information fusion methods. The terms *information fusion* and *data fusion* are typically employed as synonyms; but in some scenarios, the term *data fusion* is used for raw data (obtained directly from the sensors) and the term *information fusion* is employed to define already processed data. In this sense, the term *information fusion* implies a higher semantic level than *data fusion*. Other terms associated with data fusion that typically appear in the literature include decision fusion, data combination, data aggregation, multisensor data fusion, and sensor fusion.

Researchers in this field agree that the most accepted definition of data fusion was provided by the Joint Directors of Laboratories (JDL) workshop [1]: *“A multi-level process dealing with the association, correlation, combination of data and information from single and multiple sources to achieve refined position, identify estimates and complete and timely assessments of situations, threats and their significance.”*

Hall and Llinas [2] provided the following well-known definition of data fusion: “*data fusion techniques combine data from multiple sensors and related information from associated databases to achieve improved accuracy and more specific inferences than could be achieved by the use of a single sensor alone*.”

Briefly, we can define data fusion as a combination of multiple sources to obtain improved information; in this context, improved information means less expensive, higher quality, or more relevant information.

Data fusion techniques have been extensively employed on multisensor environments with the aim of fusing and aggregating data from different sensors; however, these techniques can also be applied to other domains, such as text processing. The goal of using data fusion in multisensor environments is to obtain a lower detection error probability and a higher reliability by using data from multiple distributed sources.

The available data fusion techniques can be classified into three nonexclusive categories: (i) data association, (ii) state estimation, and (iii) decision fusion. Because of the large number of published papers on data fusion, this paper does not aim to provide an exhaustive review of all of the studies; instead, the objective is to highlight the main steps that are involved in the data fusion framework and to review the most common techniques for each step.

The remainder of this paper continues as follows. The next section provides various classification categories for data fusion techniques. Then, Section 3 describes the most common methods for data association tasks.  Section 4 provides a review of techniques under the state estimation category. Next, the most common techniques for decision fusion are enumerated in Section 5. Finally, the conclusions obtained from reviewing the different methods are highlighted in Section 6.

## 2. Classification of Data Fusion Techniques

Data fusion is a multidisciplinary area that involves several fields, and it is difficult to establish a clear and strict classification. The employed methods and techniques can be divided according to the following criteria:

1. attending to the relations between the input data sources, as proposed by Durrant-Whyte [3]. These relations can be defined as (a) complementary, (b) redundant, or (3) cooperative data;
2. according to the input/output data types and their nature, as proposed by Dasarathy [4];
3. following an abstraction level of the employed data: (a) raw measurement, (b) signals, and (c) characteristics or decisions;
4. based on the different data fusion levels defined by the JDL;
5. Depending on the architecture type: (a) centralized, (b) decentralized, or (c) distributed.

### 2.1. Classification Based on the Relations between the Data Sources

**Figure 1:**
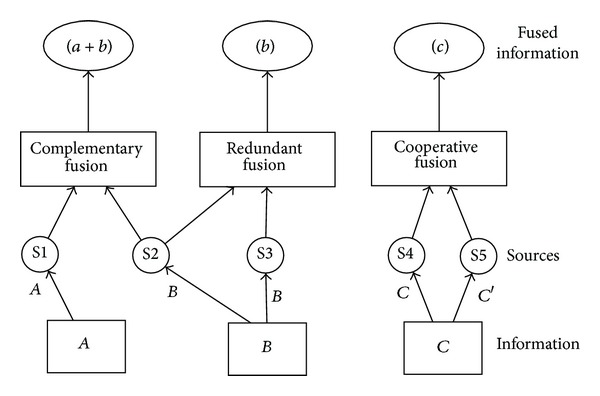
Whyte's classification based on the relations between the data sources.

Based on the relations of the sources (see Figure 1), Durrant-Whyte [3] proposed the following classification criteria:

1. complementary: when the information provided by the input sources represents different parts of the scene and could thus be used to obtain more complete global information. For example, in the case of visual sensor networks, the information on the same target provided by two cameras with different fields of view is considered complementary;
2. redundant: when two or more input sources provide information about the same target and could thus be fused to increment the confidence. For example, the data coming from overlapped areas in visual sensor networks are considered redundant;
3. cooperative: when the provided information is combined into new information that is typically more complex than the original information. For example, multi-modal (audio and video) data fusion is considered cooperative.

### 2.2. Dasarathy's Classification

**Figure 2:**
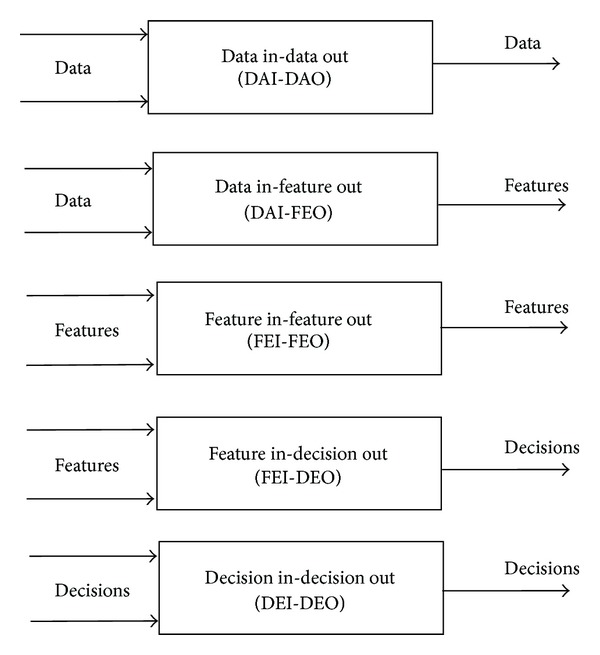
Dasarathy's classification.

One of the most well-known data fusion classification systems was provided by Dasarathy [4] and is composed of the following five categories (see Figure 2):

1. data in-data out (DAI-DAO): this type is the most basic or elementary data fusion method that is considered in classification. This type of data fusion process inputs and outputs raw data; the results are typically more reliable or accurate. Data fusion at this level is conducted immediately after the data are gathered from the sensors. The algorithms employed at this level are based on signal and image processing algorithms;
2. data in-feature out (DAI-FEO): at this level, the data fusion process employs raw data from the sources to extract features or characteristics that describe an entity in the environment;
3. feature in-feature out (FEI-FEO): at this level, both the input and output of the data fusion process are features. Thus, the data fusion process addresses a set of features with to improve, refine or obtain new features. This process is also known as feature fusion, symbolic fusion, information fusion or intermediate-level fusion;
4. feature in-decision out (FEI-DEO): this level obtains a set of features as input and provides a set of decisions as output. Most of the classification systems that perform a decision based on a sensor's inputs fall into this category of classification;
5. Decision In-Decision Out (DEI-DEO): This type of classification is also known as decision fusion. It fuses input decisions to obtain better or new decisions.

The main contribution of Dasarathy's classification is the specification of the abstraction level either as an input or an output, providing a framework to classify different methods or techniques.

### 2.3. Classification Based on the Abstraction Levels

Luo et al. [5] provided the following four abstraction levels:

1. signal level: directly addresses the signals that are acquired from the sensors;
2. pixel level: operates at the image level and could be used to improve image processing tasks;
3. characteristic: employs features that are extracted from the images or signals (i.e., shape or velocity),
4. symbol: at this level, information is represented as symbols; this level is also known as the decision level.

Information fusion typically addresses three levels of abstraction: (1) measurements, (2) characteristics, and (3) decisions. Other possible classifications of data fusion based on the abstraction levels are as follows:

1. low level fusion: the raw data are directly provided as an input to the data fusion process, which provide more accurate data (a lower signal-to-noise ratio) than the individual sources;
2. medium level fusion: characteristics or features (shape, texture, and position) are fused to obtain features that could be employed for other tasks. This level is also known as the feature or characteristic level;
3. high level fusion: this level, which is also known as decision fusion, takes symbolic representations as sources and combines them to obtain a more accurate decision. Bayesian's methods are typically employed at this level;
4. multiple level fusion: this level addresses data provided from different levels of abstraction (i.e., when a measurement is combined with a feature to obtain a decision).

### 2.4. JDL Data Fusion Classification

**Figure 3:**
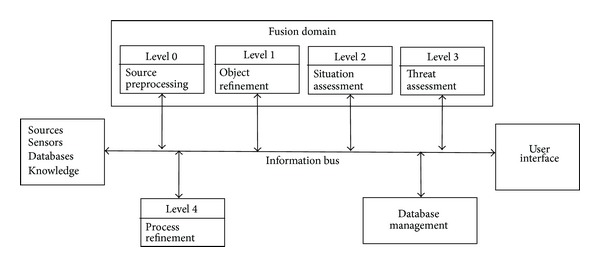
The JDL data fusion framework.

This classification is the most popular conceptual model in the data fusion community. It was originally proposed by JDL and the American Department of Defense (DoD) [1]. These organizations classified the data fusion process into five processing levels, an associated database, and an information bus that connects the five components (see Figure 3). The five levels could be grouped into two groups, low-level fusion and high-level fusion, which comprise the following components:

- sources: the sources are in charge of providing the input data. Different types of sources can be employed, such as sensors, a priori information (references or geographic data), databases, and human inputs;
- human-computer interaction (HCI): HCI is an interface that allows inputs to the system from the operators and produces outputs to the operators. HCI includes queries, commands, and information on the obtained results and alarms;
- database management system: the database management system stores the provided information and the fused results. This system is a critical component because of the large amount of highly diverse information that is stored.

In contrast, the five levels of data processing are defined as follows:

1. level 0—source preprocessing: source preprocessing is the lowest level of the data fusion process, and it includes fusion at the signal and pixel levels. In the case of text sources, this level also includes the information extraction process. This level reduces the amount of data and maintains useful information for the high-level processes;
2. level 1—object refinement: object refinement employs the processed data from the previous level. Common procedures of this level include spatio-temporal alignment, association, correlation, clustering or grouping techniques, state estimation, the removal of false positives, identity fusion, and the combining of features that were extracted from images. The output results of this stage are the object discrimination (classification and identification) and object tracking (state of the object and orientation). This stage transforms the input information into consistent data structures;
3. level 2—situation assessment: this level focuses on a higher level of inference than level 1. Situation assessment aims to identify the likely situations given the observed events and obtained data. It establishes relationships between the objects. Relations (i.e., proximity, communication) are valued to determine the significance of the entities or objects in a specific environment. The aim of this level includes performing high-level inferences and identifying significant activities and events (patterns in general). The output is a set of high-level inferences;
4. level 3—impact assessment: this level evaluates the impact of the detected activities in level 2 to obtain a proper perspective. The current situation is evaluated, and a future projection is performed to identify possible risks, vulnerabilities, and operational opportunities. This level includes (1) an evaluation of the risk or threat and (2) a prediction of the logical outcome;
5. level 4—process refinement: this level improves the process from level 0 to level 3 and provides resource and sensor management. The aim is to achieve efficient resource management while accounting for task priorities, scheduling, and the control of available resources.

High-level fusion typically starts at level 2 because the type, localization, movement, and quantity of the objects are known at that level. One of the limitations of the JDL method is how the uncertainty about previous or subsequent results could be employed to enhance the fusion process (feedback loop). Llinas et al. [6] propose several refinements and extensions to the JDL model. Blasch and Plano [7] proposed to add a new level (user refinement) to support a human user in the data fusion loop. The JDL model represents the first effort to provide a detailed model and a common terminology for the data fusion domain. However, because their roots originate in the military domain, the employed terms are oriented to the risks that commonly occur in these scenarios. The Dasarathy model differs from the JDL model with regard to the adopted terminology and employed approach. The former is oriented toward the differences among the input and output results, independent of the employed fusion method. In summary, the Dasarathy model provides a method for understanding the relations between the fusion tasks and employed data, whereas the JDL model presents an appropriate fusion perspective to design data fusion systems.

### 2.5. Classification Based on the Type of Architecture

One of the main questions that arise when designing a data fusion system is where the data fusion process will be performed. Based on this criterion, the following types of architectures could be identified:

1. centralized architecture: in a centralized architecture, the fusion node resides in the central processor that receives the information from all of the input sources. Therefore, all of the fusion processes are executed in a central processor that uses the provided raw measurements from the sources. In this schema, the sources obtain only the observationas measurements and transmit them to a central processor, where the data fusion process is performed. If we assume that data alignment and data association are performed correctly and that the required time to transfer the data is not significant, then the centralized scheme is theoretically optimal. However, the previous assumptions typically do not hold for real systems. Moreover, the large amount of bandwidth that is required to send raw data through the network is another disadvantage for the centralized approach. This issue becomes a bottleneck when this type of architecture is employed for fusing data in visual sensor networks. Finally, the time delays when transferring the information between the different sources are variable and affect the results in the centralized scheme to a greater degree than in other schemes;
2. decentralized architecture: a decentralized architecture is composed of a network of nodes in which each node has its own processing capabilities and there is no single point of data fusion. Therefore, each node fuses its local information with the information that is received from its peers. Data fusion is performed autonomously, with each node accounting for its local information and the information received from its peers. Decentralized data fusion algorithms typically communicate information using the Fisher and Shannon measurements instead of the object's state [8].
3. The main disadvantage of this architecture is the communication cost, which is *O*(*n* ^2^) at each communication step, where *n* is the number of nodes; additionally, the extreme case is considered, in which each node communicates with all of its peers. Thus, this type of architecture could suffer from scalability problems when the number of nodes is increased;
4. distributed architecture: in a distributed architecture, measurements from each source node are processed independently before the information is sent to the fusion node; the fusion node accounts for the information that is received from the other nodes. In other words, the data association and state estimation are performed in the source node before the information is communicated to the fusion node. Therefore, each node provides an estimation of the object state based on only their local views, and this information is the input to the fusion process, which provides a fused global view. This type of architecture provides different options and variations that range from only one fusion node to several intermediate fusion nodes;
5. hierarchical architecture: other architectures comprise a combination of decentralized and distributed nodes, generating hierarchical schemes in which the data fusion process is performed at different levels in the hierarchy.

In principle, a decentralized data fusion system is more difficult to implement because of the computation and communication requirements. However, in practice, there is no single best architecture, and the selection of the most appropriate architecture should be made depending on the requirements, demand, existing networks, data availability, node processing capabilities, and organization of the data fusion system.

**Figure 4:**
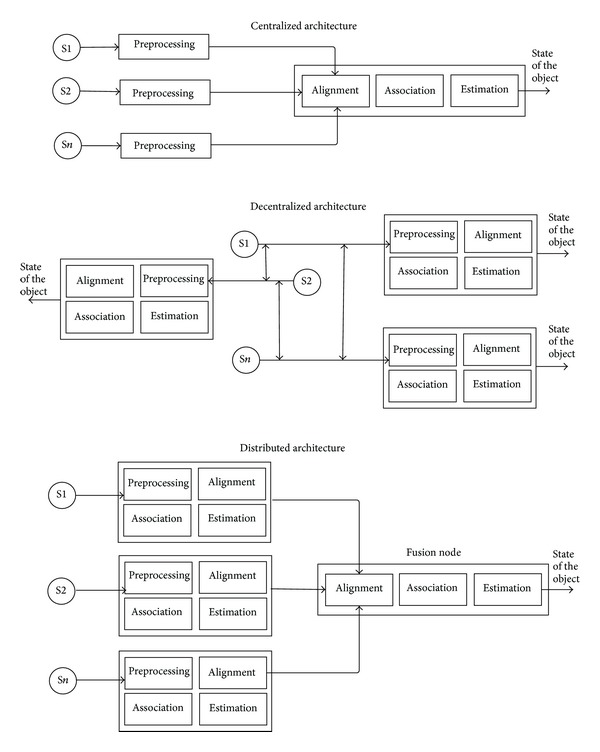
Classification based on the type of architecture.

The reader might think that the decentralized and distributed architectures are similar; however, they have meaningful differences (see Figure 4). First, in a distributed architecture, a preprocessing of the obtained measurements is performed, which provides a vector of features as a result (the features are fused thereafter). In contrast, in the decentralized architecture, the complete data fusion process is conducted in each node, and each of the nodes provides a globally fused result. Second, the decentralized fusion algorithms typically communicate information, employing the Fisher and Shannon measurements. In contrast, distributed algorithms typically share a common notion of state (position, velocity, and identity) with their associated probabilities, which are used to perform the fusion process [9]. Third, because the decentralized data fusion algorithms exchange information instead of states and probabilities, they have the advantage of easily separating old knowledge from new knowledge. Thus, the process is additive, and the associative meaning is not relevant when the information is received and fused. However, in the distributed data fusion algorithms (i.e., distributed by Kalman Filter), the state that is going to be fused is not associative, and when and how the fused estimates are computed is relevant. Nevertheless, in contrast to the centralized architectures, the distributed algorithms reduce the necessary communication and computational costs because some tasks are computed in the distributed nodes before data fusion is performed in the fusion node.

## 3. Data Association Techniques

**Figure 5:**
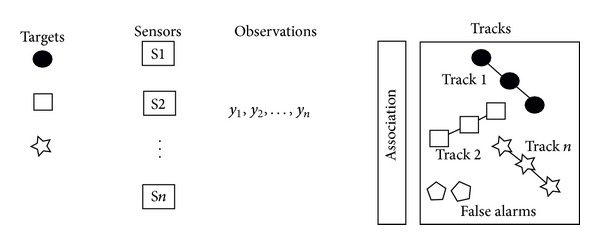
Conceptual overview of the data association process from multiple sensors and multiple targets. It is necessary to establish the set of observations over time from the same object that forms a track.

The data association problem must determine the set of measurements that correspond to each target (see Figure 5). Let us suppose that there are *O* targets that are being tracked by only one sensor in a cluttered environment (by a cluttered environment, we refer to an environment that has several targets that are to close each other). Then, the data association problem can be defined as follows:

- each sensor's observation is received in the fusion node at discrete time intervals;
- the sensor might not provide observations at a specific interval;
- some observations are noise, and other observations originate from the detected target;
- for any specific target and in every time interval, we do not know (a priori) the observations that will be generated by that target.

Therefore, the goal of data association is to establish the set of observations or measurements that are generated by the same target over time. Hall and Llinas [2] provided the following definition of data association: *“The process of assign and compute the weights that relates the observations or tracks* (A track can be defined as an ordered set of points that follow a path and are generated by the same target.) *from one set to the observation of tracks of another set.”*

As an example of the complexity of the data association problem, if we take a frame-to-frame association and assume that *M* possible points could be detected in all *n* frames, then the number of possible sets is (*M*!)^*n*−1^. Note that from all of these possible solutions, only one set establishes the true movement of the *M* points.

Data association is often performed before the state estimation of the detected targets. Moreover, it is a key step because the estimation or classification will behave incorrectly if the data association phase does not work coherently. The data association process could also appear in all of the fusion levels, but the granularity varies depending on the objective of each level.

In general, an exhaustive search of all possible combinations grows exponentially with the number of targets; thus, the data association problem becomes NP complete. The most common techniques that are employed to solve the data association problem are presented in the following sections (from Sections 3.1 to 3.7).

### 3.1. Nearest Neighbors and *K*-Means

Nearest neighbor (NN) is the simplest data association technique. NN is a well-known clustering algorithm that selects or groups the most similar values. How close the one measurement is to another depends on the employed distance metric and typically depends on the threshold that is established by the designer. In general, the employed criteria could be based on (1) an absolute distance, (2) the Euclidean distance, or (3) a statistical function of the distance.

NN is a simple algorithm that can find a feasible (approximate) solution in a small amount of time. However, in a cluttered environment, it could provide many pairs that have the same probability and could thus produce undesirable error propagation [10]. Moreover, this algorithm has poor performance in environments in which false measurements are frequent, which are in highly noisy environments.

All neighbors use a similar technique, in which all of the measurements inside a region are included in the tracks.

*K*-Means [11] method is a well-known modification of the NN algorithm. *K*-Means divides the dataset values into *K* different clusters. *K*-Means algorithm finds the *best* localization of the cluster centroids, where *best* means a centroid that is in the center of the data cluster. *K*-Means is an iterative algorithm that can be divided into the following steps:

1. obtain the input data and the number of desired clusters (*K*);
2. randomly assign the centroid of each cluster;
3. match each data point with the centroid of each cluster;
4. move the cluster centers to the centroid of the cluster;
5. if the algorithm does not converge, return to step (3).

*K*-Means is a popular algorithm that has been widely employed; however, it has the following disadvantages:

- the algorithm does not always find the optimal solution for the cluster centers;
- the number of clusters must be known a priori and one must assume that this number is the optimum;
- the algorithm assumes that the covariance of the dataset is irrelevant or that it has been normalized already.

There are several options for overcoming these limitations. For the first one, it is possible to execute the algorithm several times and obtain the solution that has less variance. For the second one, it is possible to start with a low value of *K* and increment the values of *K* until an adequate result is obtained. The third limitation can be easily overcome by multiplying the data with the inverse of the covariance matrix.

Many variations have been proposed to Lloyd's basic *K*-Means algorithm [11], which has a computational upper bound cost of *O*(*K* ^*n*^), where *n* is the number of input points and *K* is the number of desired clusters. Some algorithms modify the initial cluster assignments to improve the separations and reduce the number of iterations. Others introduce soft or multinomial clustering assignments using fuzzy logic, probabilistic, or the Bayesian techniques. However, most of the previous variations still must perform several iterations through the data space to converge to a reasonable solution. This issue becomes a major disadvantage in several real-time applications. A new approach that is based on having a large (but still affordable) number of cluster candidates compared to the desired *K* clusters is currently gaining attention. The idea behind this computational model is that the algorithm builds a good sketch of the original data while reducing the dimensionality of the input space significantly. In this manner, a weighted *K*-Means can be applied to the large candidate clusters to derive a good clustering of the original data. Using this idea, [12] presented an efficient and scalable *K*-Means algorithm that is based on random projections. This algorithm requires only one pass through the input data to build the clusters. More specifically, if the input data distribution holds some separability requirements, then the number of required candidate clusters grows only according to *O*(log⁡ *n*), where *n* is the number of observations in the original data. This salient feature makes the algorithm scalable in terms of both the memory and computational requirements.

### 3.2. Probabilistic Data Association

The probabilistic data association (PDA) algorithm was proposed by Bar-Shalom and Tse [13] and is also known as the modified filter of all neighbors. This algorithm assigns an association probability to each hypothesis from a valid measurement of a target. A valid measurement refers to the observation that falls in the validation gate of the target at that time instant. The validation gate, *γ*, which is the center around the predicted measurements of the target, is used to select the set of basic measurements and is defined as

(1) $$\begin{array}{c} \gamma \geq {\left(Z\left(k\right)-\hat{z}\left(k\;|\;k-1\right)\right)}^{T}S^{-1}\left(k\right)\left(z\left(k\right)-z\left(k\;|\;k-1\right)\right), \end{array}$$

where *K* is the temporal index, *S*(*k*) is the covariance gain, and *γ* determines the gating or window size. The set of valid measurements at time instant *k* is defined as

(2) $$\begin{array}{c} Z\left(k\right)=z_{i}\left(k\right),\;i=1,\ldots ,m_{k}, \end{array}$$

where *z*_*i*_(*k*) is the *i*-measurement in the validation region at time instant *k*. We give the standard equations of the PDA algorithm next. For the state prediction, consider

(3) $$\begin{array}{c} \hat{x}\left(k\;|\;k-1\right)=F\left(k-1\right)\hat{x}\left(k-1\;|\;k-1\right), \end{array}$$

where *F*(*k* − 1) is the transition matrix at time instant *k* − 1. To calculate the measurement prediction, consider

(4) $$\begin{array}{c} \hat{z}\left(k\;|\;k-1\right)=H\left(k\right)\hat{x}\left(k\;|\;k-1\right), \end{array}$$

where *H*(*k*) is the linearization measurement matrix. To compute the gain or the innovation of the *i*-measurement, consider

(5) $$\begin{array}{c} v_{i}\left(k\right)=z_{i}\left(k\right)-\hat{z}\left(k\;|\;k-1\right). \end{array}$$

To calculate the covariance prediction, consider

(6) $$\begin{array}{c} \hat{P}\left(k\;|\;k-1\right)=F\left(k-1\right)\hat{P}\left(k-1\;|\;k-1\right)F{\left(k-1\right)}^{T}+Q\left(k\right), \end{array}$$

where *Q*(*k*) is the process noise covariance matrix. To compute the innovation covariance (*S*) and the Kalman gain (*K*)

(7) $$\begin{array}{c} S\left(k\right)=H\left(k\right)\hat{P}\left(k\;|\;k-1\right)H{\left(k\right)}^{T}+R,\\ K\left(k\right)=\hat{P}\left(k\;|\;k-1\right)H{\left(k\right)}^{T}S{\left(k\right)}^{-1}. \end{array}$$

To obtain the covariance update in the case in which the measurements originated by the target are known, consider

(8) $$\begin{array}{c} P^{0}\left(k\;|\;k\right)=\hat{P}\left(k\;|\;k-1\right)-K\left(k\right)S\left(k\right)K{\left(k\right)}^{T}. \end{array}$$

The total update of the covariance is computed as

(9) $$\begin{array}{c} v\left(k\right)=\overset{m_{k}}{\underset{i=1}{\sum }}\beta_{i}\left(k\right)v_{i}\left(k\right),\\ P\left(k\right)=K\left(k\right)\left[\overset{m_{k}}{\underset{i=1}{\sum }}\left(\beta_{i}\left(k\right)v_{i}\left(k\right)v_{i}{\left(k\right)}^{T}\right)-v\left(k\right)v{\left(k\right)}^{T}\right]K^{T}\left(k\right), \end{array}$$

where *m*_*k*_ is the number of valid measurements in the instant *k*. The equation to update the estimated state, which is formed by the position and velocity, is given by

(10) $$\begin{array}{c} \hat{x}\left(k\;|\;k\right)=\hat{x}\left(k\;|\;k-1\right)+K\left(k\right)v\left(k\right). \end{array}$$

Finally, the association probabilities of PDA are as follows:

(11) $$\begin{array}{c} \beta_{i}\left(k\right)=\frac{p_{i}\left(k\right)}{\sum_{i=0}^{m_{k}}p_{i}\left(k\right)}, \end{array}$$

where

(12) $$\begin{array}{c} p_{i}\left(k\right)=\{\begin{array}{cc} \frac{{\left(2\Pi \right)}^{M/2}\lambda \sqrt{\left|S_{i}\left(k\right)\right|}\left(1-P_{d}P_{g}\right)}{P_{d}} & \text{if}\;i=0\\ \exp\left[\frac{-1}{2}v^{T}\left(k\right)S^{-1}\left(k\right)v\left(k\right)\right] & \text{if}\;i\neq 0\\ 0 & \text{in}\;\text{other}\;\text{cases}, \end{array} \end{array}$$

where *M* is the dimension of the measurement vector, *λ* is the density of the clutter environment, *P*_*d*_ is the detection probability of the correct measurement, and *P*_*g*_ is the validation probability of a detected value.

In the PDA algorithm, the state estimation of the target is computed as a weighted sum of the estimated state under all of the hypotheses. The algorithm can associate different measurements to one specific target. Thus, the association of the different measurements to a specific target helps PDA to estimate the target state, and the association probabilities are used as weights. The main disadvantages of the PDA algorithm are the following:

- loss of tracks: because PDA ignores the interference with other targets, it sometimes could wrongly classify the closest tracks. Therefore, it provides a poor performance when the targets are close to each other or crossed;
- the suboptimal Bayesian approximation: when the source of information is uncertain, PDA is the suboptimal Bayesian approximation to the association problem;
- one target: PDA was initially designed for the association of one target in a low-cluttered environment. The number of false alarms is typically modeled with the Poisson distribution, and they are assumed to be distributed uniformly in space. PDA behaves incorrectly when there are multiple targets because the false alarm model does not work well;
- track management: because PDA assumes that the track is already established, algorithms must be provided for track initialization and track deletion.

PDA is mainly good for tracking targets that do not make abrupt changes in their movement patterns. PDA will most likely lose the target if it makes abrupt changes in its movement patterns.

### 3.3. Joint Probabilistic Data Association

Joint probabilistic data association (JPDA) is a suboptimal approach for tracking multiple targets in cluttered environments [14]. JPDA is similar to PDA, with the difference that the association probabilities are computed using all of the observations and all of the targets. Thus, in contrast to PDA, JPDA considers various hypotheses together and combines them. JPDA determines the probability *β*_*i*_^*t*^(*k*) that measurement *i* is originated from target *t*, accounting for the fact that under this hypothesis, the measurement cannot be generated by other targets. Therefore, for a known number of targets, it evaluates the different options of the measurement-target association (for the most recent set of measurements) and combines them into the corresponding state estimation. If the association probability is known, then the Kalman filter updating equation of the track *t* can be written as

(13) $$\begin{array}{c} {\hat{x}}^{t}\left(k\;|\;k\right)={\hat{x}}^{t}\left(k\;|\;k-1\right)+K\left(k\right){\overset{-}{v}}^{t}\left(k\right), \end{array}$$

where ${\hat{x}}^{t}(k|k)$ and ${\hat{x}}^{t}(k|k-1)$ are the estimation and prediction of target *t*, and *K*(*k*) is the filter gain. The weighted sum of the residuals associated with the observation *m*(*k*) of target *t* is as follows:

(14) $$\begin{array}{c} {\overset{-}{v}}^{t}\left(k\right)=\overset{m(k)}{\underset{i=1}{\sum }}\beta_{i}^{t}\left(k\right)v_{i}^{t}\left(k\right), \end{array}$$

where *v*_*i*_^*t*^ = *z*_*i*_(*k*) − *Hx* ^*t*^(*k* | *k* − 1). Therefore, this method incorporates all of the observations (inside the neighborhood of the target's predicted position) to update the estimated position by using a posterior probability that is a weighted sum of residuals.

The main restrictions of JPDA are the following:

- a measurement cannot come from more than one target;
- two measurements cannot be originated by the same target (at one time instant);
- the sum of all of the measurements' probabilities that are assigned to one target must be 1: ∑_*i*=0_^*m*(*k*)^ *β*_*i*_^*t*^(*k*) = 1.

The main disadvantages of JPDA are the following:

- it requires an explicit mechanism for track initialization. Similar to PDA, JPDA cannot initialize new tracks or remove tracks that are out of the observation area;
- JPDA is a computationally expensive algorithm when it is applied in environments that have multiple targets because the number of hypotheses is incremented exponentially with the number of targets.

In general, JPDA is more appropriate than MHT in situations in which the density of false measurements is high (i.e., sonar applications).

### 3.4. Multiple Hypothesis Test

The underlying idea of the multiple hypothesis test (MHT) is based on using more than two consecutive observations to make an association with better results. Other algorithms that use only two consecutive observations have a higher probability of generating an error. In contrast to PDA and JPDA, MHT estimates all of the possible hypotheses and maintains new hypotheses in each iteration.

MHT was developed to track multiple targets in cluttered environments; as a result, it combines the data association problem and tracking into a unified framework, becoming an estimation technique as well. The Bayes rule or the Bayesian networks are commonly employed to calculate the MHT hypothesis. In general, researchers have claimed that MHT outperforms JPDA for the lower densities of false positives. However, the main disadvantage of MHT is the computational cost when the number of tracks or false positives is incremented. Pruning the hypothesis tree using a window could solve this limitation.

The Reid [15] tracking algorithm is considered the standard MHT algorithm, but the initial integer programming formulation of the problem is due to Morefield [16]. MHT is an iterative algorithm in which each iteration starts with a set of correspondence hypotheses. Each hypothesis is a collection of disjoint tracks, and the prediction of the target in the next time instant is computed for each hypothesis. Next, the predictions are compared with the new observations by using a distance metric. The set of associations established in each hypothesis (based on a distance) introduces new hypotheses in the next iteration. Each new hypothesis represents a new set of tracks that is based on the current observations.

Note that each new measurement could come from (i) a new target in the visual field of view, (ii) a target being tracked, or (iii) noise in the measurement process. It is also possible that a measurement is not assigned to a target because the target disappears, or because it is not possible to obtain a target measurement at that time instant.

MHT maintains several correspondence hypotheses for each target in each frame. If the hypothesis in the instant *k* is represented by *H*(*k*) = [*h*_*l*_(*k*), *k* = 1,…, *n*], then the probability of the hypothesis *h*_*l*_(*k*) could be represented recursively using the Bayes rule as follows:

(15) P(hl(k) ∣ Z(k))=P(hg(k−1),ai(k) ∣ Z(k))=1cP(Z(k) ∣ hg(k−1),ai(k)) ∗P(ai(k) ∣ hg(k−1))∗P(hg(k−1)),

where *h*_*g*_(*k* − 1) is the hypothesis *g* of the complete set until the time instant *k* − 1; *a*_*i*_(*k*) is the *i*th possible association of the track to the object; *Z*(*k*) is the set of detections of the current frame, and *c* is a normal constant.

The first term on the right side of the previous equation is the likelihood function of the measurement set *Z*(*k*) given the joint likelihood and current hypothesis. The second term is the probability of the association hypothesis of the current data given the previous hypothesis *h*_*g*_(*k* − 1). The third term is the probability of the previous hypothesis from which the current hypothesis is calculated.

The MHT algorithm has the ability to detect a new track while maintaining the hypothesis tree structure. The probability of a true track is given by the Bayes decision model as

(16) $$\begin{array}{c} P\left(\lambda \;|\;Z\right)=\frac{P\left(Z\;|\;\lambda \right)*P_{\circ }\left(\lambda \right)}{P\left(Z\right)}, \end{array}$$

where *P*(*Z* | *λ*) is the probability of obtaining the set of measurements *Z* given *λ*, *P*_∘_(*λ*) is the a priori probability of the source signal, and *P*(*Z*) is the probability of obtaining the set of detections *Z*.

MHT considers all of the possibilities, including both the track maintenance and the initialization and removal of tracks in an integrated framework. MHT calculates the possibility of having an object after the generation of a set of measurements using an exhaustive approach, and the algorithm does not assume a fixed number of targets. The key challenge of MHT is the effective hypothesis management. The baseline MHT algorithm can be extended as follows: (i) use the hypothesis aggregation for missed targets births, cardinality tracking, and closely spaced objects; (ii) apply a multistage MHT for improving the performance and robustness in challenging settings; and (iii) use a feature-aided MHT for extended object surveillance.

The main disadvantage of this algorithm is the computational cost, which grows exponentially with the number of tracks and measurements. Therefore, the practical implementation of this algorithm is limited because it is exponential in both time and memory.

With the aim of reducing the computational cost, [17] presented a probabilistic MHT algorithm in which the associations are considered to be random variables that are statistically independent and in which performing an exhaustive search enumeration is avoided. This algorithm is known as PMHT. The PMHT algorithm assumes that the number of targets and measurements is known. With the same goal of reducing the computational cost, [18] presented an efficient implementation of the MHT algorithm. This implementation was the first version to be applied to perform tracking in visual environments. They employed the Murty [19] algorithm to determine the best set of *k* hypotheses in polynomial time, with the goal of tracking the points of interest.

MHT typically performs the tracking process by employing only one characteristic, commonly the position. The Bayesian combination to use multiple characteristics was proposed by Liggins II et al. [20].

A linear-programming-based relaxation approach to the optimization problem in MHT tracking was proposed independently by Coraluppi et al. [21] and Storms and Spieksma [22]. Joo and Chellappa [23] proposed an association algorithm for tracking multiple targets in visual environments. Their algorithm is based on in MHT modification in which a measurement can be associated with more than one target, and several targets can be associated with one measurement. They also proposed a combinatorial optimization algorithm to generate the best set of association hypotheses. Their algorithm always finds the best hypothesis, in contrast to other models, which are approximate. Coraluppi and Carthel [24] presented a generalization of the MHT algorithm using a recursion over hypothesis classes rather than over a single hypothesis. This work has been applied in a special case of the multi-target tracking problem, called cardinality tracking, in which they observed the number of sensor measurements instead of the target states.

### 3.5. Distributed Joint Probabilistic Data Association

The distributed version of the joint probabilistic data association (JPDA-D) was presented by Chang et al. [25]. In this technique, the estimated state of the target (using two sensors) after being associated is given by

(17) E{x ∣ Z1,Z2}=∑j=0m1∑ l=0m2E{x ∣ χj1,χl2,Z1,Z2}∗P{χj1,χl2 ∣ Z1,Z2},

where *m*_*i*_, *i* = 1,2, is the last set of measurements of sensor 1 and 2, *Z* ^*i*^, *i* = 1,2, is the set of accumulative data, and *χ* is the association hypothesis. The first term of the right side of the equation is calculated from the associations that were made earlier. The second term is computed from the individual association probabilities as follows:

(18) P(χj1,χl2 ∣ Z1,Z2)=∑x1∑x2=P(χ1,χ2 ∣ Z1,Z2)ω^j1(χ1)ω^l2(χ2),P(χ1,χ2 ∣ Z1,Z2)=1cP(χ1 ∣ Z1)P(χ2 ∣ Z2)γ(χ1,χ2),

where *χ* ^*i*^ are the joint hypotheses involving all of the measurements and all of the objectives, and ${\hat{\omega }}_{j}^{i}(\chi^{i})$ are the binary indicators of the measurement-target association. The additional term *γ*(*χ* ^1^, *χ* ^2^) depends on the correlation of the individual hypothesis and reflects the localization influence of the current measurements in the joint hypotheses.

These equations are obtained assuming that communication exists after every observation, and there are only approximations in the case in which communication is sporadic and when a substantial amount of noise occurs. Therefore, this algorithm is a theoretical model that has some limitations in practical applications. 

### 3.6. Distributed Multiple Hypothesis Test

The distributed version of the MHT algorithm (MHT-D) [26, 27] follows a similar structure as the JPDA-D algorithm. Let us assume the case in which one node must fuse two sets of hypotheses and tracks. If the hypotheses and track sets are represented by *H* ^*i*^(*Z* ^*i*^) and *T* ^*i*^(*Z* ^*i*^) with *i* = 1,2, the hypothesis probabilities are represented by *λ*_*j*_^*i*^; and the state distribution of the tracks (*τ*_*j*_^*i*^) is represented by *P*(*λ*_*j*_^*i*^) and *P*(*x* | *Z* ^*i*^, *τ*_*j*_^*i*^); then, the maximum available information in the fusion node is *Z* = *Z* ^1^ ∪ *Z* ^2^. The data fusion objective of the MHT-D is to obtain the set of hypotheses *H*(*Z*), the set of tracks *T*(*Z*), the hypothesis probabilities *P*(*λ* | *Z*), and the state distribution *p*(*x* | *Z*, *τ*) for the observed data.

The MHT-D algorithm is composed of the following steps:

(1) hypothesis formation: for each hypothesis pair *λ*_*j*_^1^ and *λ*_*k*_^2^, which could be fused, a track *τ* is formed by associating the pair of tracks *τ*_*j*_^1^ and *τ*_*k*_^2^, where each pair comes from one node and could originate from the same target. The final result of this stage is a set of hypotheses denoted by *H*(*Z*) and the fused tracks *T*(*Z*);
(2) hypothesis evaluation: in this stage, the association probability of each hypothesis and the estimated state of each fused track are obtained. The distributed estimation algorithm is employed to calculate the likelihood of the possible associations and the obtained estimations at each specific association. Using the information model, the probability of each fused hypothesis is given by
  (19) $$\begin{array}{c} P\left(\lambda \;|\;Z\right)=C^{-1}\underset{j\in J}{\prod }P{\left(\lambda^{\left(j\right)}\;|\;Z^{\left(j\right)}\right)}^{\alpha (j)}\underset{\tau \in \lambda }{\prod }L\left(\tau \;|\;Z\right), \end{array}$$

where *C* is a normalizing constant, and *L*(*τ* | *Z*) is the likelihood of each hypothesis pair.

The main disadvantage of the MHT-D is the high computational cost that is in the order of *O*(*n* ^*M*^), where *n* is the number of possible associations and *M* is the number of variables to be estimated. 

### 3.7. Graphical Models

Graphical models are a formalism for representing and reasoning with probabilities and independence. A graphical model represents a conditional decomposition of the joint probability. A graphical model can be represented as a graph in which the nodes denote random variables; the edges denote the possible dependence between the random variables, and the plates denote the replication of a substructure, with the appropriate indexing of the relevant variables. The graph captures the joint distribution over the random variables, which can be decomposed into a product of factors that each depend on only a subset of variables. There are two major classes of graphical models: (i) the Bayesian networks [28], which are also known as the directed graphical models, and (ii) the Markov random fields, which are also known as undirected graphical models. The directed graphical models are useful for expressing causal relationships between random variables, whereas undirected models are better suited for expressing soft constraints between random variables. We refer the reader to the book of Koller and Friedman [29] for more information on graphical models.

A framework based on graphical models can solve the problem of distributed data association in synchronized sensor networks with overlapped areas and where each sensor receives noisy measurements; this solution was proposed by Chen et al. [30, 31]. Their work is based on graphical models that are used to represent the statistical dependence between random variables. The data association problem is treated as an inference problem and solved by using the max-product algorithm [32]. Graphical models represent statistical dependencies between variables as graphs, and the max-product algorithm converges when the graph is a tree structure. Moreover, the employed algorithm could be implemented in a distributed manner by exchanging messages between the source nodes in parallel. With this algorithm, if each sensor has *n* possible combinations of associations and there are *M* variables to be estimated, it has a complexity of *O*(*n* ^2^ *M*), which is reasonable and less than the *O*(*n* ^*M*^) complexity of the MHT-D algorithm. However, aspecial attention must be given to the correlated variables when building the graphical model. 

## 4. State Estimation Methods

State estimation techniques aim to determine the state of the target under movement (typically the position) given the observation or measurements. State estimation techniques are also known as tracking techniques. In their general form, it is not guaranteed that the target observations are relevant, which means that some of the observations could actually come from the target and others could be only noise. The state estimation phase is a common stage in data fusion algorithms because the target's observation could come from different sensors or sources, and the final goal is to obtain a global target state from the observations.

The estimation problem involves finding the values of the vector state (e.g., position, velocity, and size) that fits as much as possible with the observed data. From a mathematical perspective, we have a set of redundant observations, and the goal is to find the set of parameters that provides the best fit to the observed data. In general, these observations are corrupted by errors and the propagation of noise in the measurement process. State estimation methods fall under level 1 of the JDL classification and could be divided into two broader groups:

1. linear dynamics and measurements: here, the estimation problem has a standard solution. Specifically, when the equations of the object state and the measurements are linear, the noise follows the Gaussian distribution, and we do not refer to it as a clutter environment; in this case, the optimal theoretical solution is based on the Kalman filter;
2. nonlinear dynamics: the state estimation problem becomes difficult, and there is not an analytical solution to solve the problem in a general manner. In principle, there are no practical algorithms available to solve this problem satisfactorily.

Most of the state estimation methods are based on control theory and employ the laws of probability to compute a vector state from a vector measurement or a stream of vector measurements. Next, the most common estimation methods are presented, including maximum likelihood and maximum posterior (Section 4.1), the Kalman filter (Section 4.2), particle filter (Section 4.3), the distributed Kalman filter (Section 4.4), distributed particle filter (Section 4.5) and, covariance consistency methods (Section 4.6).

### 4.1. Maximum Likelihood and Maximum Posterior

The maximum likelihood (ML) technique is an estimation method that is based on probabilistic theory. Probabilistic estimation methods are appropriate when the state variable follows an unknown probability distribution [33]. In the context of data fusion, *x* is the state that is being estimated, and *z* = (*z*(1),…, *z*(*k*)) is a sequence of *k* previous observations of *x*. The likelihood function *λ*(*x*) is defined as a probability density function of the sequence of *z* observations given the true value of the state *x*. Consider

(20) $$\begin{array}{c} \lambda \left(x\right)=p\left(z\;|\;x\right). \end{array}$$

The ML estimator finds the value of *x* that maximizes the likelihood function:

(21) $$\begin{array}{c} \hat{x}\left(k\right)=\arg\underset{x}{\max}\;p\left(z\;|\;x\right), \end{array}$$

which can be obtained from the analytical or empirical models of the sensors. This function expresses the probability of the observed data. The main disadvantage of this method in practice is that it requires the analytical or empirical model of the sensor to be known to provide the prior distribution and compute the likelihood function. This method can also systematically underestimate the variance of the distribution, which leads to a bias problem. However, the bias of the ML solution becomes less significant as the number *N* of data points increases and is equal to the true variance of the distribution that generated the data at the limit *N* → *∞*.

The maximum posterior (MAP) method is based on the Bayesian theory. It is employed when the parameter *x* to be estimated is the output of a random variable that has a known probability density function *p*(*x*). In the context of data fusion, *x* is the state that is being estimated and *z* = (*z*(1),…, *z*(*k*)) is a sequence of *k* previous observations of *x*. The MAP estimator finds the value of *x* that maximizes the posterior probability distribution as follows:

(22) $$\begin{array}{c} \hat{x}\left(k\right)=\arg\underset{x}{\max}\;p\left(x\;|\;z\right). \end{array}$$

Both methods (ML and MAP) aim to find the most likely value for the state *x*. However, ML assumes that *x* is a fixed but an unknown point from the parameter space, whereas MAP considers *x* to be the output of a random variable with a known a priori probability density function. Both of these methods are equivalent when there is no a priori information about *x*, that is, when there are only observations.

### 4.2. The Kalman Filter

The Kalman filter is the most popular estimation technique. It was originally proposed by Kalman [34] and has been widely studied and applied since then. The Kalman filter estimates the state *x* of a discrete time process governed by the following space-time model:

(23) $$\begin{array}{c} x\left(k+1\right)=\Phi \left(k\right)x\left(k\right)+G\left(k\right)u\left(k\right)+w\left(k\right) \end{array}$$

with the observations or measurements *z* at time *k* of the state *x* represented by

(24) $$\begin{array}{c} z\left(k\right)=H\left(k\right)x\left(k\right)+v\left(k\right), \end{array}$$

where Φ(*k*) is the state transition matrix, *G*(*k*) is the input matrix transition, *u*(*k*) is the input vector, *H*(*k*) is the measurement matrix, and *w* and *v* are the random Gaussian variables with zero mean and covariance matrices of *Q*(*k*) and *R*(*k*), respectively. Based on the measurements and on the system parameters, the estimation of *x*(*k*), which is represented by $\hat{x}(k)$, and the prediction of *x*(*k* + 1), which is represented by $\hat{x}(k+1|k)$, are given by the following:

(25) $$\begin{array}{c} \hat{x}\left(k\right)=\hat{x}\left(k\;|\;k+1\right)+K\left(k\right)\left[z\left(k\right)-H\left(k\right)\hat{x}\left(k\;|\;k-1\right)\right],\\ \hat{x}\left(k+1\;|\;k\right)=\Phi \left(k\right)\hat{x}\left(k\;|\;k\right)+G\left(k\right)u\left(k\right), \end{array}$$

respectively, where *K* is the filter gain determined by

(26) K(k)=P(k ∣ k−1)HT(k) ×[H(k)P(k ∣ k−1)HT(k)+R(k)]−1,

where *P*(*k* | *k* − 1) is the prediction covariance matrix and can be determined by

(27) $$\begin{array}{c} P\left(k+1\;|\;k\right)=\Phi \left(k\right)P\left(k\right)\Phi^{T}\left(k\right)+Q\left(k\right) \end{array}$$

with

(28) $$\begin{array}{c} P\left(k\right)=P\left(k\;|\;k-1\right)-K\left(k\right)H\left(k\right)P\left(k\;|\;k-1\right). \end{array}$$

The Kalman filter is mainly employed to fuse low-level data. If the system could be described as a linear model and the error could be modeled as the Gaussian noise, then the recursive Kalman filter obtains optimal statistical estimations [35]. However, other methods are required to address nonlinear dynamic models and nonlinear measurements. The modified Kalman filter known as the extended Kalman filter (EKF) is an optimal approach for implementing nonlinear recursive filters [36]. The EKF is one of the most often employed methods for fusing data in robotic applications. However, it has some disadvantages because the computations of the Jacobians are extremely expensive. Some attempts have been made to reduce the computational cost, such as linearization, but these attempts introduce errors in the filter and make it unstable.

The unscented Kalman filter (UKF) [37] has gained popularity, because it does not have the linearization step and the associated errors of the EKF [38]. The UKF employs a deterministic sampling strategy to establish the minimum set of points around the mean. This set of points captures the true mean and covariance completely. Then, these points are propagated through nonlinear functions, and the covariance of the estimations can be recuperated. Another advantage of the UKF is its ability to be employed in parallel implementations.

### 4.3. Particle Filter

Particle filters are recursive implementations of the sequential Monte Carlo methods [39]. This method builds the posterior density function using several random samples called particles. Particles are propagated over time with a combination of sampling and resampling steps. At each iteration, the sampling step is employed to discard some particles, increasing the relevance of regions with a higher posterior probability. In the filtering process, several particles of the same state variable are employed, and each particle has an associated weight that indicates the quality of the particle. Therefore, the estimation is the result of a weighted sum of all of the particles. The standard particle filter algorithm has two phases: (1) the predicting phase and (2) the updating phase. In the predicting phase, each particle is modified according to the existing model and accounts for the sum of the random noise to simulate the noise effect. Then, in the updating phase, the weight of each particle is reevaluated using the last available sensor observation, and particles with lower weights are removed. Specifically, a generic particle filter comprises the following steps.

(1) Initialization of the particles:
  (i) let *N* be equal to the number of particles;
  (ii) *X* ^(*i*)^(1) = [*x*(1), *y*(1), 0,0]^*T*^ for *i* = 1,…, *N*.
(2) Prediction step:
  (i) for each particle *i* = 1,…, *N*, evaluate the state (*k* + 1 | *k*) of the system using the state at time instant *k* with the noise of the system at time *k*. Consider
    (29) X^(i)(k+1 ∣ k)=F(k)X^(i)(k) +(cauchy-distribution-noise)(k),
    where *F*(*k*) is the transition matrix of the system.
(3) Evaluate the particle weight. For each particle *i* = 1,…, *N*:
  (i) compute the predicted observation state of the system using the current predicted state and the noise at instant *k*. Consider
    (30) z^(i)(k+1 ∣ k)=H(k+1)X^(i)(k+1 ∣ k) +(gaussian-measurement-noise)(k+1);
  (ii) compute the likelihood (weights) according to the given distribution. Consider
    (31) $$\begin{array}{c} \text{likelihoo}{\text{d}}^{\left(i\right)}=N\left({\hat{z}}^{\left(i\right)}\left(k+1\;|\;k\right);z^{\left(i\right)}\left(k+1\right),\text{var}\right); \end{array}$$
  (iii) normalize the weights as follows
    (32) $$\begin{array}{c} {\tilde{w}}^{\left(i\right)}=\frac{\text{likelihoo}{\text{d}}^{\left(i\right)}}{\sum_{j=1}^{N}\text{likelihoo}{\text{d}}^{\left(j\right)}}. \end{array}$$
(4) Resampling/Selection: multiply particles with higher weights and remove those with lower weights. The current state must be adjusted using the computed weights of the new particles.
  (i) Compute the cumulative weights. Consider
    (33) $$\begin{array}{c} \text{Cum}\;\text{W}{\text{t}}^{\left(i\right)}=\overset{i}{\underset{j=1}{\sum }}{\tilde{w}}^{\left(j\right)}. \end{array}$$
  (ii) Generate uniform distributed random variables from *U* ^(*i*)^ ~ *W*(0,1) with the number of steps equal to the number of particles.
  (iii) Determine which particles should be multiplied and which ones removed.
(5) Propagation phase:
  (i) incorporate the new values of the state after the resampling of instant *k* to calculate the value at instant *k* + 1. Consider
    (34) $$\begin{array}{c} {\hat{x}}^{\left(1:N\right)}\left(k+1\;|\;k+1\right)=\hat{x}\left(k+1\;|\;k\right); \end{array}$$
  (ii) compute the posterior mean. Consider
    (35) $$\begin{array}{c} \hat{x}\left(k+1\right)=\text{mean}\left[x^{i}\left(k+1\;|\;k+1\right)\right],\;i=1,\ldots ,N; \end{array}$$
  (iii) repeat steps 2 to 5 for each time instant.

Particle filters are more flexible than the Kalman filters and can cope with nonlinear dependencies and non-Gaussian densities in the dynamic model and in the noise error. However, they have some disadvantages. A large number of particles are required to obtain a small variance in the estimator. It is also difficult to establish the optimal number of particles in advance, and the number of particles affects the computational cost significantly. Earlier versions of particle filters employed a fixed number of particles, but recent studies have started to use a dynamic number of particles [40]. 

### 4.4. The Distributed Kalman Filter

The distributed Kalman filter requires a correct clock synchronization between each source, as demonstrated in [41]. In other words, to correctly use the distributed Kalman filter, the clocks from all of the sources must be synchronized. This synchronization is typically achieved through using protocols that employ a shared global clock, such as the network time protocol (NTP). Synchronization problems between clocks have been shown to have an effect on the accuracy of the Kalman filter, producing inaccurate estimations [42].

If the estimations are consistent and the cross covariance is known (or the estimations are uncorrelated), then it is possible to use the distributed Kalman filters [43]. However, the cross covariance must be determined exactly, or the observations must be consistent.

We refer the reader to Liggins II et al. [20] for more details about the Kalman filter in a distributed and hierarchical architecture. 

### 4.5. Distributed Particle Filter

Distributed particle filters have gained attention recently [44–46]. Coates [45] used a distributed particle filter to monitor an environment that could be captured by the Markovian state-space model, involving nonlinear dynamics and observations and non-Gaussian noise.

In contrast, earlier attempts to solve out-of-sequence measurements using particle filters are based on regenerating the probability density function to the time instant of the out-of-sequence measurement [47]. In a particle filter, this step requires a large computational cost, in addition to the necessary space to store the previous particles. To avoid this problem, Orton and Marrs [48] proposed to store the information on the particles at each time instant, saving the cost of recalculating this information. This technique is close to optimal, and when the delay increases, the result is only slightly affected [49]. However, it requires a very large amount of space to store the state of the particles at each time instant. 

### 4.6. Covariance Consistency Methods: Covariance Intersection/Union

Covariance consistency methods (intersection and union) were proposed by Uhlmann [43] and are general and fault-tolerant frameworks for maintaining covariance means and estimations in a distributed network. These methods do not comprise estimation techniques; instead, they are similar to an estimation fusion technique. The distributed Kalman filter requirement of independent measurements or known cross-covariances is not a constraint with this method. 

#### 4.6.1. Covariance Intersection

If the Kalman filter is employed to combine two estimations, (*a*_1_, *A*_1_) and (*a*_2_, *A*_2_), then it is assumed that the joint covariance is in the following form:

(36) $$\begin{array}{c} \left[\begin{array}{cc} A_{1} & X\\ X^{T} & A_{2} \end{array}\right], \end{array}$$

where the cross-covariance *X* should be known exactly so that the Kalman filter can be applied without difficulty. Because the computation of the cross-covariances is computationally intensive, Uhlmann [43] proposed the covariance intersection (CI) algorithm.

Let us assume that a joint covariance *M* can be defined with the diagonal blocks *M*_*A*_1__ > *A*_1_ and *M*_*A*_2__ > *A*_2_. Consider

(37) $$\begin{array}{c} M⩾\left[\begin{array}{cc} A_{1} & X\\ X^{T} & A_{2} \end{array}\right] \end{array}$$

for every possible instance of the unknown cross-covariance *X*; then, the components of the matrix *M* could be employed in the Kalman filter equations to provide a fused estimation (*c*, *C*) that is considered consistent. The key point of this method relies on generating a joint covariance matrix *M* that can represent a useful fused estimation (in this context, *useful* refers to something with a lower associated uncertainty). In summary, the CI algorithm computes the joint covariance matrix *M*, where the Kalman filter provides the best fused estimation (*c*, *C*) with respect to a fixed measurement of the covariance matrix (i.e., the minimum determinant).

Specific covariance criteria must be established because there is not a specific minimum joint covariance in the order of the positive semidefinite matrices. Moreover, the joint covariance is the basis of the formal analysis of the CI algorithm; the actual result is a nonlinear mixture of the information stored on the estimations being fused, following the following equation.

(38) $$\begin{array}{c} C={\left(w_{1}H_{1}^{T}A_{1}^{-1}H_{1}+w_{2}H_{2}^{T}A_{2}^{-1}H_{2}+\cdots +w_{n}H_{n}^{T}A_{n}^{-1}H_{n}\right)}^{-1},\\ c=C{\left(w_{1}H_{1}^{T}A_{1}^{-1}a_{1}+w_{2}H_{2}^{T}A_{2}^{-1}a_{2}+\cdots +w_{n}H_{n}^{T}A_{n}^{-1}a_{n}\right)}^{-1}, \end{array}$$

where *H*_*i*_ is the transformation of the fused state-space estimation to the space of the estimated state *i*. The values of *w* can be calculated to minimize the covariance determinant using convex optimization packages and semipositive matrix programming. The result of the CI algorithm has different characteristics compared to the Kalman filter. For example, if two estimations are provided (*a*, *A*) and (*b*, *B*) and their covariances are equal *A* = *B*, since the Kalman filter is based on the statistical independence assumption, it produces a fused estimation with covariance *C* = (1/2)*A*. In contrast, the CI method does not assume independence and, thus, must be consistent even in the case in which the estimations are completely correlated, with the estimated fused covariance *C* = *A*. In the case of estimations where *A* < *B*, the CI algorithm does not provide information about the estimation (*b*, *B*); thus, the fused result is (*a*, *A*).

Every joint-consistent covariance is sufficient to produce a fused estimation, which guarantees consistency. However, it is also necessary to guarantee a lack of divergence. Divergence is avoided in the CI algorithm by choosing a specific measurement (i.e., the determinant), which is minimized in each fusion operation. This measurement represents a non-divergence criterion, because the size of the estimated covariance according to this criterion would not be incremented.

The application of the CI method guarantees consistency and nondivergence for every sequence of mean and covariance-consistent estimations. However, this method does not work well when the measurements to be fused are inconsistent. 

#### 4.6.2. Covariance Union

CI solves the problem of correlated inputs but not the problem of inconsistent inputs (*inconsistent inputs* refer to different estimations, each of which has a high accuracy (small variance) but also a large difference from the states of the others); thus, the covariance union (CU) algorithm was proposed to solve the latter [43]. CU addresses the following problem: two estimations (*a*_1_, *A*_1_) and (*a*_2_, *A*_2_) relate to the state of an object and are mutually inconsistent from one another. This issue arises when the difference between the average estimations is larger than the provided covariance. Inconsistent inputs can be detected using the Mahalanobis distance [50] between them, which is defined as

(39) $$\begin{array}{c} M_{d}={\left(a_{1}-a_{2}\right)}^{T}{\left(A_{1}+A_{2}\right)}^{-1}\left(a_{1}-a_{2}\right), \end{array}$$

and detecting whether this distance is larger than a given threshold.

The Mahalanobis distance accounts for the covariance information to obtain the distance. If the difference between the estimations is high but their covariance is also high, the Mahalanobis distance yields a small value. In contrast, if the difference between the estimations is small and the covariances are small, it could produce a larger distance value. A high Mahalanobis distance could indicate that the estimations are inconsistent; however, it is necessary to have a specific threshold established by the user or learned automatically.

The CU algorithm aims to solve the following problem: let us suppose that a filtering algorithm provides two observations with mean and covariance (*a*_1_, *A*_1_) and (*a*_2_, *A*_2_), respectively. It is known that one of the observations is correct and the other is erroneous. However, the identity of the correct estimation is unknown and cannot be determined. In this situation, if both estimations are employed as an input to the Kalman filter, there will be a problem, because the Kalman filter only guarantees a consistent output if the observation is updated with a measurement consistent with both of them. In the specific case, in which the measurements correspond to the same object but are acquired from two different sensors, the Kalman filter can only guarantee that the output is consistent if it is consistent with both separately. Because it is not possible to know which estimation is correct, the only way to combine the two estimations rigorously is to provide an estimation (*u*, *U*) that is consistent with both estimations and to obey the following properties:

(40) $$\begin{array}{c} U⪖A_{1}+\left(u-a_{1}\right){\left(u-A_{1}\right)}^{T},\\ U⪖A_{2}+\left(u-a_{2}\right){\left(u-A_{2}\right)}^{T}, \end{array}$$

where some measurement of the matrix size *U* (i.e., the determinant) is minimized.

In other words, the previous equations indicate that if the estimation (*a*_1_, *A*_1_) is consistent, then the translation of the vector *a*_1_ to *u* requires to increase the covariance by the sum of a matrix at least as big as the product of (*u* − *a*_1_) in order to be consistent. The same situation applies to the measurement (*a*_2_, *A*_2_) in order to be consistent.

A simple strategy is to choose the mean of the estimation as the input value of one of the measurements (*u* = *a*_1_). In this case, the value of *U* must be chosen, such that the estimation is consistent with the worst case (the correct measurement is *a*_2_). However, it is possible to assign *u* an intermediate value between *a*_1_ and *a*_2_ to decrease the value of *U*. Therefore, the CU algorithm establishes the mean fused value *u* that has the least covariance *U* but is sufficiently large for the two measurements (*a*_1_ and *a*_2_) for consistency.

Because the matrix inequalities presented in previous equations are convex, convex optimization algorithms must be employed to solve them. The value of *U* can be computed with the iterative method described by Julier et al. [51]. The obtained covariance could be significantly larger than any of the initial covariances and is an indicator of the existing uncertainty between the initial estimations. One of the advantages of the CU method arises from the fact that the same process could be easily extended to *N* inputs. 

## 5. Decision Fusion Methods

A decision is typically taken based on the knowledge of the perceived situation, which is provided by many sources in the data fusion domain. These techniques aim to make a high-level inference about the events and activities that are produced from the detected targets. These techniques often use symbolic information, and the fusion process requires to reason while accounting for the uncertainties and constraints. These methods fall under level 2 (situation assessment) and level 4 (impact assessment) of the JDL data fusion model.

### 5.1. The Bayesian Methods

Information fusion based on the Bayesian inference provides a formalism for combining evidence according to the probability theory rules. Uncertainty is represented using the conditional probability terms that describe beliefs and take on values in the interval [0,1], where zero indicates a complete lack of belief and one indicates an absolute belief. The Bayesian inference is based on the Bayes rule as follows:

(41) $$\begin{array}{c} P\left(Y\;|\;X\right)=\frac{P\left(X\;|\;Y\right)P\left(Y\right)}{P\left(X\right)}, \end{array}$$

where the posterior probability, *P*(*Y* | *X*), represents the belief in the hypothesis *Y* given the information *X*. This probability is obtained by multiplying the a priori probability of the hypothesis *P*(*Y*) by the probability of having *X* given that *Y* is true, *P*(*X* | *Y*). The value *P*(*X*) is used as a normalizing constant. The main disadvantage of the Bayesian inference is that the probabilities *P*(*X*) and *P*(*X* | *Y*) must be known. To estimate the conditional probabilities, Pan et al. [52] proposed the use of NNs, whereas Coué et al. [53] proposed the Bayesian programming.

Hall and Llinas [54] described the following problems associated with Bayesian inference.

- Difficulty in establishing the value of a priori probabilities.
- Complexity when there are multiple potential hypotheses and a substantial number of events that depend on the conditions.
- The hypothesis should be mutually exclusive.
- Difficulty in describing the uncertainty of the decisions.

### 5.2. The Dempster-Shafer Inference

The Dempster-Shafer inference is based on the mathematical theory introduced by Dempster [55] and Shafer [56], which generalizes the Bayesian theory. The Dempster-Shafer theory provides a formalism that could be used to represent incomplete knowledge, updating beliefs, and a combination of evidence and allows us to represent the uncertainty explicitly [57].

A fundamental concept in the Dempster-Shafer reasoning is the frame of discernment, which is defined as follows. Let Θ = {*θ*_1_, *θ*_2_,…, *θ*_*N*_} be the set of all possible states that define the system, and let Θ be exhaustive and mutually exclusive due to the system being only in one state *θ*_*i*_ ∈ Θ, where 1⪕*i*⪕*N*. The set Θ is called a frame of discernment, because its elements are employed to discern the current state of the system.

The elements of the set 2^Θ^ are called hypotheses. In the Dempster-Shafer theory, based on the evidence *E*, a probability is assigned to each hypothesis *H* ∈ 2^Θ^ according to the basic assignment of probabilities or the mass function *m* : 2^Θ^ → [0.1], which satisfies

(42) $$\begin{array}{c} m\left(\emptyset \right)=0. \end{array}$$

Thus, the mass function of the empty set is zero. Furthermore, the mass function of a hypothesis is larger than or equal to zero for all of the hypotheses. Consider

(43) $$\begin{array}{c} m\left(H\right)\geq 0,\;\forall H\in 2^{\Theta }. \end{array}$$

The sum of the mass function of all the hypotheses is one. Consider

(44) $$\begin{array}{c} \underset{H\in 2^{\Theta }}{\sum }m\left(H\right)=1. \end{array}$$

To express incomplete beliefs in a hypothesis *H*, the Dempster-Shafer theory defines the belief function bel : 2^Θ^ → [0,1] over Θ as

(45) $$\begin{array}{c} \text{bel}\left(H\right)=\underset{A\subseteq H}{\sum }m\left(A\right), \end{array}$$

where bel(*∅*) = 0, and bel(Θ) = 1. The doubt level in *H* can be expressed in terms of the belief function by

(46) $$\begin{array}{c} \text{dou}\left(H\right)=\text{bel}\left(\neg H\right)=\underset{A\subseteq \neg H}{\sum }m\left(A\right). \end{array}$$

To express the plausibility of each hypothesis, the function pl : 2^Θ^ → [0,1] over Θ is defined as

(47) $$\begin{array}{c} \text{pl}\left(H\right)=1-\text{dou}\left(H\right)=\underset{A\cap H=\emptyset }{\sum }m\left(A\right). \end{array}$$

Intuitive plausibility indicates that there is less uncertainty in hypothesis *H* if it is more plausible. The confidence interval [bel(*H*), pl(*H*)] defines the true belief in hypothesis *H*. To combine the effects of the two mass functions *m*_1_ and *m*_2_, the Dempster-Shafer theory defines a rule *m*_1_ ⊕ *m*_2_ as

(48) $$\begin{array}{c} m_{1}⊕m_{2}\left(\emptyset \right)=0,\\ m_{1}⊕m_{2}\left(H\right)=\frac{\sum_{X\cap Y=H}m_{1}\left(X\right)m_{2}\left(Y\right)}{1-\sum_{X\cap Y=\emptyset }m_{1}\left(X\right)m_{2}\left(Y\right)}. \end{array}$$

In contrast to the Bayesian inference, a priori probabilities are not required in the Dempster-Shafer inference, because they are assigned at the instant that the information is provided. Several studies in the literature have compared the use of the Bayesian inference and the Dempster-Shafer inference, such as [58–60]. Wu et al. [61] used the Dempster-Shafer theory to fuse information in context-aware environments. This work was extended in [62] to dynamically modify the associated weights to the sensor measurements. Therefore, the fusion mechanism is calibrated according to the recent measurements of the sensors (in cases in which the ground-truth is available). In the military domain [63], the Dempster-Shafer reasoning is used with the a priori information stored in a database for classifying military ships. Morbee et al. [64] described the use of the Dempster-Shafer theory to build 2D occupancy maps from several cameras and to evaluate the contribution of subsets of cameras to a specific task. Each task is the observation of an event of interest, and the goal is to assess the validity of a set of hypotheses that are fused using the Dempster-Shafer theory. 

### 5.3. Abductive Reasoning

Abductive reasoning, or inferring the best explanation, is a reasoning method in which a hypothesis is chosen under the assumption that in case it is true, it explains the observed event most accurately [65]. In other words, when an event is observed, the abduction method attempts to find the best explanation.

In the context of probabilistic reasoning, abductive inference finds the posterior ML of the system variables given some observed variables. Abductive reasoning is more a reasoning pattern than a data fusion technique. Therefore, different inference methods, such as NNs [66] or fuzzy logic [67], can be employed.

### 5.4. Semantic Methods

Decision fusion techniques that employ semantic data from different sources as an input could provide more accurate results than those that rely on only single sources. There is a growing interest in techniques that automatically determine the presence of semantic features in videos to solve the semantic gap [68].

Semantic information fusion is essentially a scheme in which raw sensor data are processed such that the nodes exchange only the resultant semantic information. Semantic information fusion typically covers two phases: (i) building the knowledge and (ii) pattern matching (inference). The first phase (typically offline) incorporates the most appropriate knowledge into semantic information. Then, the second phase (typically online or in real-time) fuses relevant attributes and provides a semantic interpretation of the sensor data [69–71].

Semantic fusion could be viewed as an idea for integrating and translating sensor data into formal languages. Therefore, the obtained resulting language from the observations of the environment is compared with similar languages that are stored in the database. The key of this strategy is that similar behaviors represented by formal languages are also semantically similar. This type of method provides savings in the cost of transmission, because the nodes need only transmit the formal language structure instead of the raw data. However, a known set of behaviors must be stored in a database in advance, which might be difficult in some scenarios.

## 6. Conclusions

This paper reviews the most popular methods and techniques for performing data/information fusion. To determine whether the application of data/information fusion methods is feasible, we must evaluate the computational cost of the process and the delay introduced in the communication. A centralized data fusion approach is theoretically optimal when there is no cost of transmission and there are sufficient computational resources. However, this situation typically does not hold in practical applications.

The selection of the most appropriate technique depends on the type of the problem and the established assumptions of each technique. Statistical data fusion methods (e.g., PDA, JPDA, MHT, and Kalman) are optimal under specific conditions [72]. First, the assumption that the targets are moving independently and the measurements are normally distributed around the predicted position typically does not hold. Second, because the statistical techniques model all of the events as probabilities, they typically have several parameters and a priori probabilities for false measurements and detection errors that are often difficult to obtain (at least in an optimal sense). For example, in the case of the MHT algorithm, specific parameters must be established that are nontrivial to determine and are very sensitive [73]. In contrast, statistical methods that optimize over several frames are computationally intensive, and their complexity typically grows exponentially with the number of targets. For example, in the case of particle filters, tracking several targets can be accomplished jointly as a group or individually. If several targets are tracked jointly, the necessary number of particles grows exponentially. Therefore, in practice, it is better to perform tracking on them individually, with the assumption that targets do not interact between the particles.

In contrast to centralized systems, the distributed data fusion methods introduce some challenges in the data fusion process, such as (i) spatial and temporal alignments of the information, (ii) out-of-sequence measurements, and (iii) data correlation reported by Castanedo et al. [74, 75]. The inherent redundancy of the distributed systems could be exploited with distributed reasoning techniques and cooperative algorithms to improve the individual node estimations reported by Castanedo et al. [76]. In addition to the previous studies, a new trend based on the geometric notion of a low-dimensional manifold is gaining attention in the data fusion community. An example is the work of Davenport et al. [77], which proposes a simple model that captures the correlation between the sensor observations by matching the parameter values for the different obtained manifolds.
